# Best vitelliform macular dystrophy in a large Brazilian family

**DOI:** 10.1186/s40942-019-0156-0

**Published:** 2019-01-30

**Authors:** Carolina Pádua Rocha de Souza, Luiz Guilherme Marchesi Mello, Fernando Gomez, Eduardo Morizot

**Affiliations:** 1 Benjamin Constant Institute (IBC), Av. Pasteur, 350 - Urca, Rio de Janeiro, Rio de Janeiro 22290-240 Brazil; 2 Federal University of Espírito Santo (UFES), Vitória, Espírito Santo Brazil

**Keywords:** Autofluorescence, Fluorescein angiography, Optical coherence tomography, Retina, Vitelliform macular dystrophy

## Abstract

**Background:**

To describe the clinical and multimodal imaging findings of a Brazilian family with Best vitelliform macular dystrophy.

**Methods:**

A retrospective chart review of a Brazilian family was conducted and complementary fundus images (color photography, autofluorescence, fluorescein angiography and optical coherence tomography) were analyzed.

**Results:**

Seven patients had typical macular lesions at different stages of Best vitelliform macular dystrophy. Electrooculography was performed in two of them and showed abnormal Arden ratio. The pedigree strongly suggests an autosomal dominant inheritance. Low visual acuity was mainly associated with advanced age, retinal pigment epithelium atrophy, and photoreceptors damage. However, yellow subretinal deposits were evidenced in patients with better visual acuity.

**Conclusion:**

We present the largest case series of a Brazilian family with Best vitelliform macular dystrophy. Multimodal imaging analysis is important to determine retinal abnormalities. Retinal pigment epithelium atrophy and loss of photoreceptors outer segments seem to be a late but important finding related to severe visual acuity impairment.

## Background

Best vitelliform macular dystrophy (BVMD), also known as Best disease, is a rare progressive autosomal dominant macular degeneration with variable penetrance and expression. Ocular lesions are associated with mutations in *BEST1* (*VMD2*) gene, located on chromosome 11q13 [1]. Bestrophin-1 is a basolateral transmembrane protein of the retinal pigment epithelium (RPE), encoded by *BEST1* gene, and function as an intracellular calcium-activated chloride channel and an anion channel. Therefore, *BEST1* mutation alters ions and fluids transport in RPE, RPE microvilli and RPE-photoreceptor outer segment (POS) interaction/adhesiveness. Consequently, serous RPE-neuroretinal detachment occurs and impaired phagocytosis of subretinal debris leads to abnormal accumulation of lipofuscin within RPE cells with photoreceptor damage in the initial phases of the disease. The subretinal lipofuscin and cellular debris create the classical vitelliform “egg-yolk” lesion, leading to visual impairment [2]. The age of onset varies even in identical mutations and within the same family, with a median around 15 years [3, 4]. Based on clinical exam findings, BVMD is generally divided into five stages [5]: normal macula or subtle RPE changes (Stage I, or Previtelliform), vitelliform “egg-yolk” lesions (Stage II, or Vitelliform), pseudohypopyon due to layering/gravitation of the lipofuscin (Stage III, or Pseudohypopyon), disruption of the yellowish material and RPE mobilization giving an appearance of “scrambled-egg” (Stage IV, or Vitelliruptive), and absorption of subretinal fluid/deposits leaving RPE atrophy (Stage V, or Atrophic). Severe visual acuity impairment is usually observed in late stages of the disease when foveal atrophy/fibrosis occurs [5]. Complementary exams, such as fundus photography, autofluorescence (FAF), fluorescein angiography (FA), and optical coherence tomography (OCT) are essential to monitor patients with BVMD or a family history of this disease. They allow early diagnosis, detection of macular functional and anatomical complications, and may be used in the planning of future therapeutic interventions [3, 6].

In this study, we report the multimodal fundus imaging of fourteen eyes with different stages of BVMD. To our knowledge, this is the largest case series of a Brazilian family with Best vitelliform macular dystrophy.

## Methodology

**Fig. 1:**
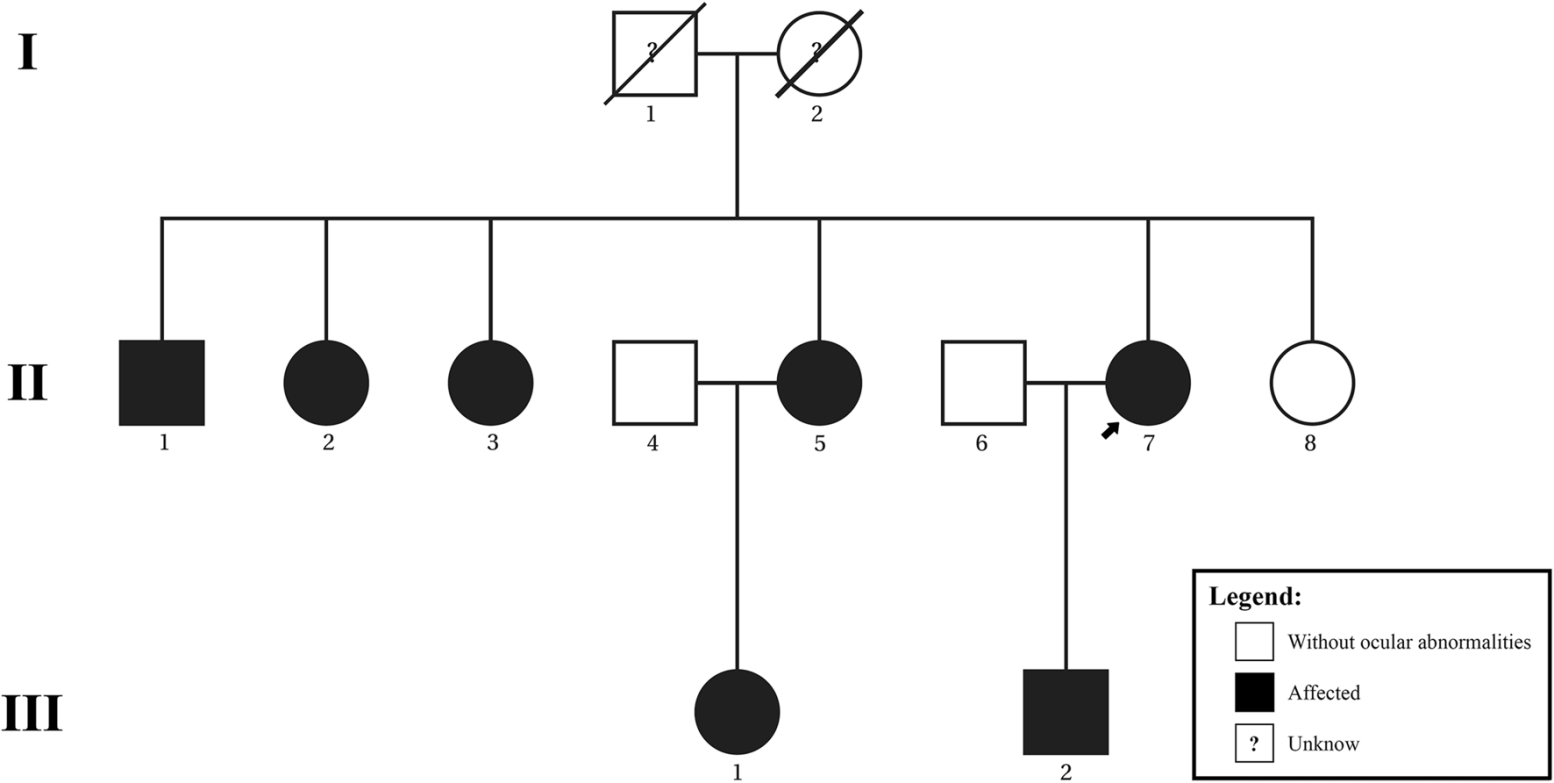
Pedigree structure of the studied Brazilian family. Examined patients include proband (II-7), siblings (II-1, II-2, II-3, II-5, and II-8), niece (III-1), and daughter (III-2)

This is a descriptive and retrospective study of seven patients with BVMD from the same family, identified through a review of medical records at the Retina Department of Benjamin Constant Institute (IBC), Rio de Janeiro, Brazil. This study was approved by the Institutional Ethics Committee (Number 143774/2018; CAAE 03437118.8.0000.5246). All patients (14 eyes) underwent a complete ophthalmologic examination including best-corrected visual acuity, slit-lamp biomicroscopy, and Goldmann applanation tonometry. Fundus photography, FAF, FA, and OCT were performed in respectively ten, eight, six, and twelve eyes, with a digital fundus camera (Canon CX-1; Canon Inc., Tokyo) and the Heidelberg Spectralis OCT (Heidelberg Engineering, Heidelberg, Germany). A pedigree was formulated (Fig. 1).

## Results

**Fig. 2:**
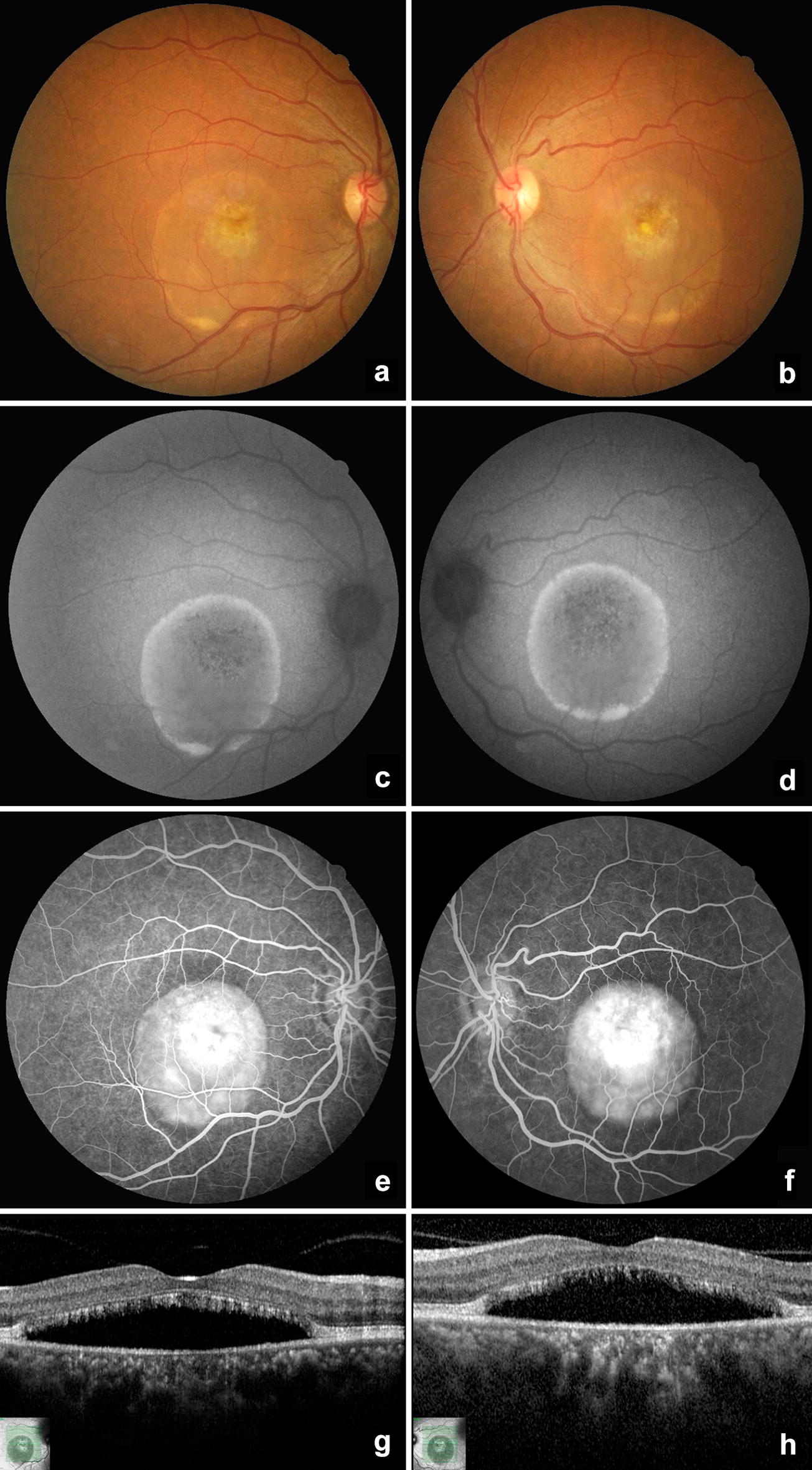
Color fundus photography (**a** and** b**), fundus autofluorescence (**c** and** d**), fluorescein angiography (**e** and** f**) and optical coherence tomography (**g** and** h**) of the proband (II-7) in the “pseudohypopyon” stage. The yellowish vitelliform material displaced inferiorly and a thin ring of lipofuscin accumulation is seen as a hyperautofluorescent margin of the neurosensorial retinal detachment. The central area is hypoautofluorescent and hyperfluorescent due to pigment epithelial atrophy (window defect)

The proband of the present study is a 54-year-old woman (Fig. 1, II-7), who came for a routine ophthalmological evaluation. She had an unremarkable previous medical and ocular history. However, she reported a family history of four siblings with visual problems (unknown diagnosis). On ocular examination, the best-corrected visual acuity was 20/25 in both eyes with hyperopic spherical equivalent (right eye: + 1.00; left eye: + 1.50). The ocular motility, pupillary reactions, and slit-lamp biomicroscopy of the anterior segment were normal. Fundoscopy revealed bilateral macular yellowish deposits (Fig. 2a, b). FAF showed a small rounded subfoveal hyperautofluorescent material, surrounded by hypoautofluorescence corresponding to RPE atrophy and a circular hyper-autofluorescence ring in both eyes (Fig. 2c, d). FA and OCT evidenced serous macular detachment and deposits of foveal round-shaped subretinal material suggestive of lipofuscin and debris of photoreceptors in both eyes (Fig. 2e–h). Electrooculography (EOG) showed a reduced Arden ratio in both eyes and confirmed the hypothesis of BVMD.

**Fig. 3:**
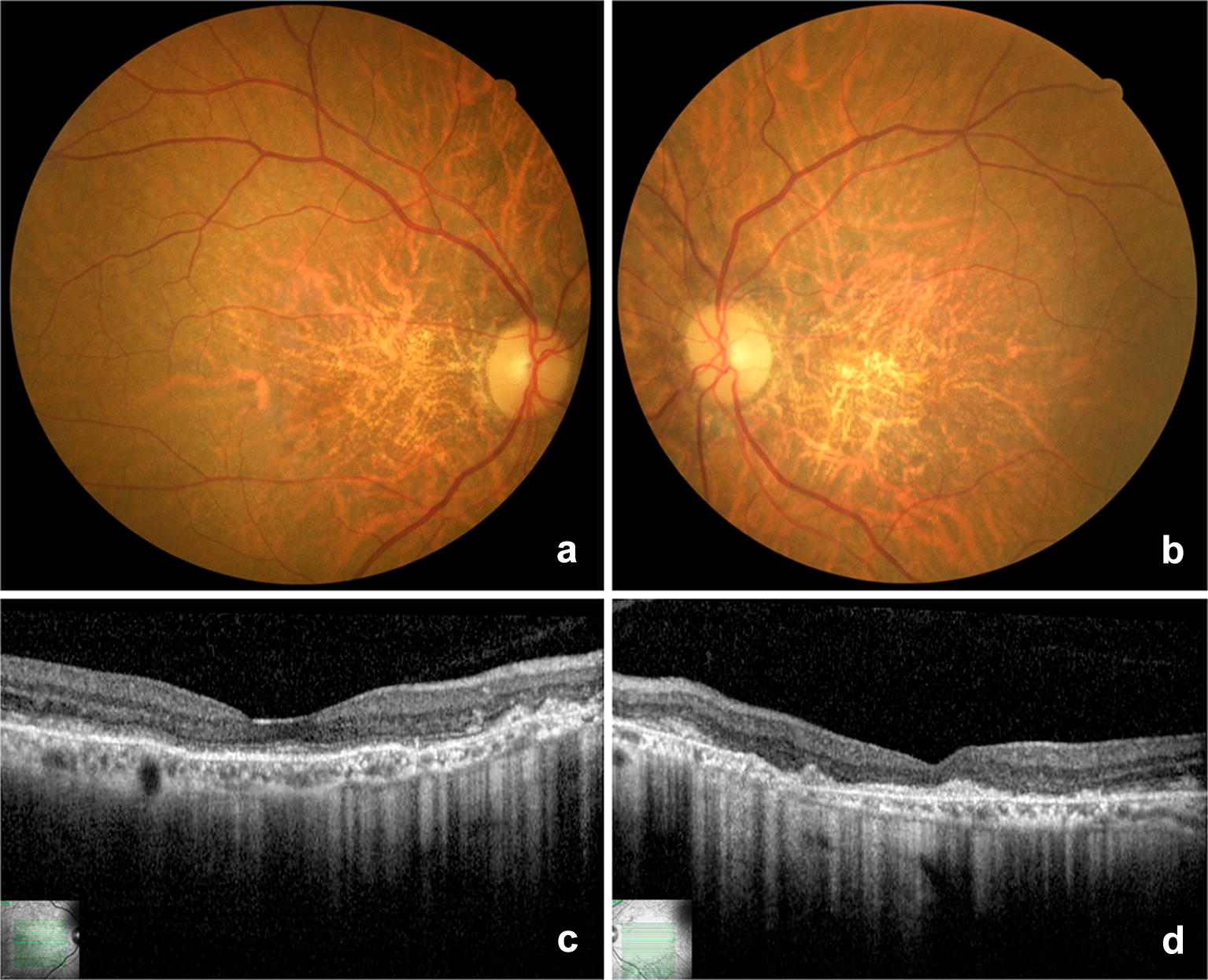
Color fundus photography (**a** and **b**) and optical coherence tomography (**c** and **d**) of the patient II-1 in the “atrophic” stage. Right and left fovea are slightly yellowish due to retinal pigment epithelium atrophy and residual dispersed subretinal fibrotic material. Optical coherence tomography reveals diffuse loss of photoreceptor in both eyes, but with right eye foveolar sparing

**Fig. 4:**
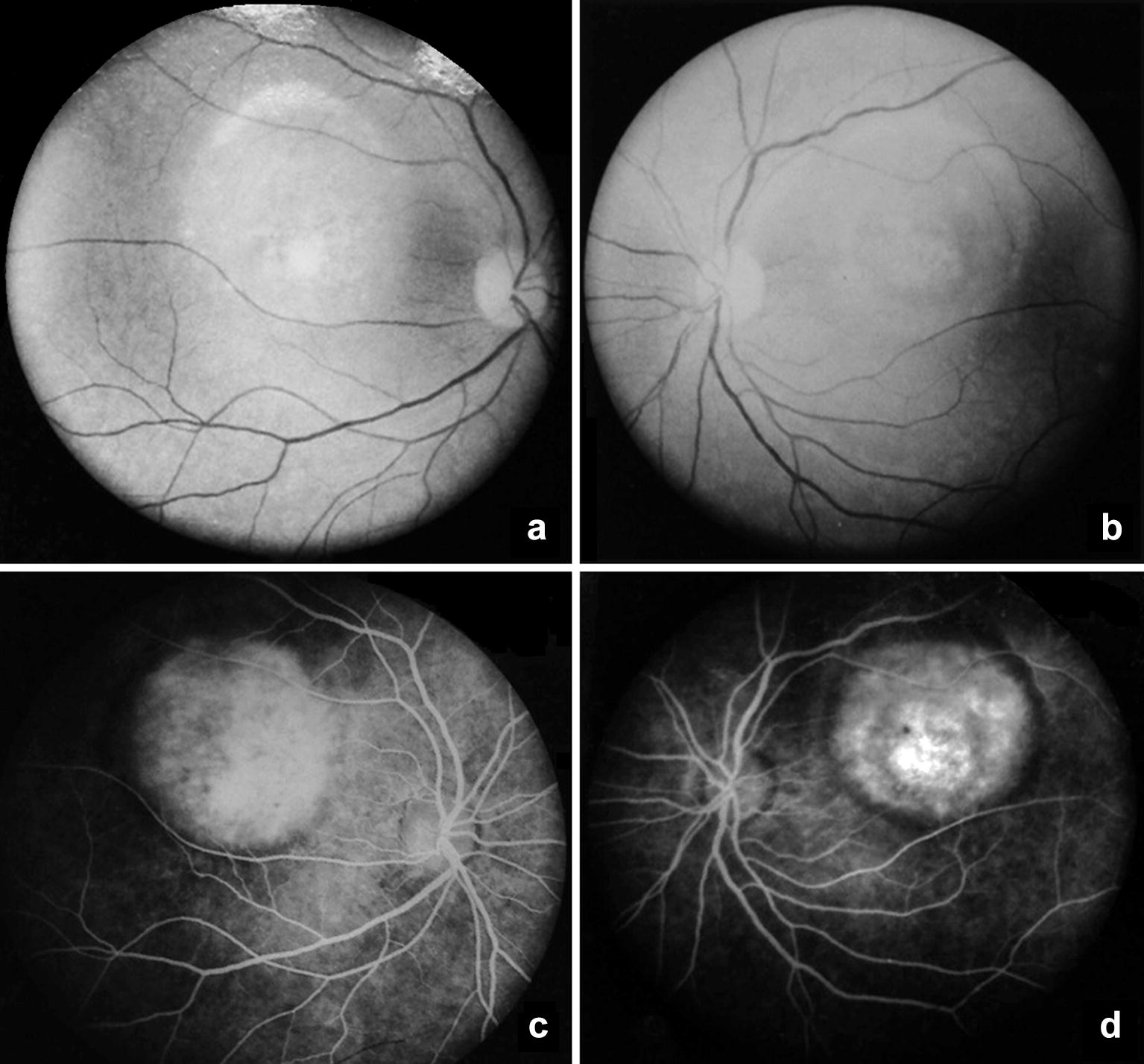
Red-free light fundus photography (**a** and **b**) and fluorescein angiography (**c** and **d**) of patient II-2 in the “atrophic” stage. Red-free frames show the central white rounded macular lesion. Fluorescein angiography shows foveal hyperfluorescence without leakage due to due to pigment epithelial atrophy (window defect)

**Fig. 5:**
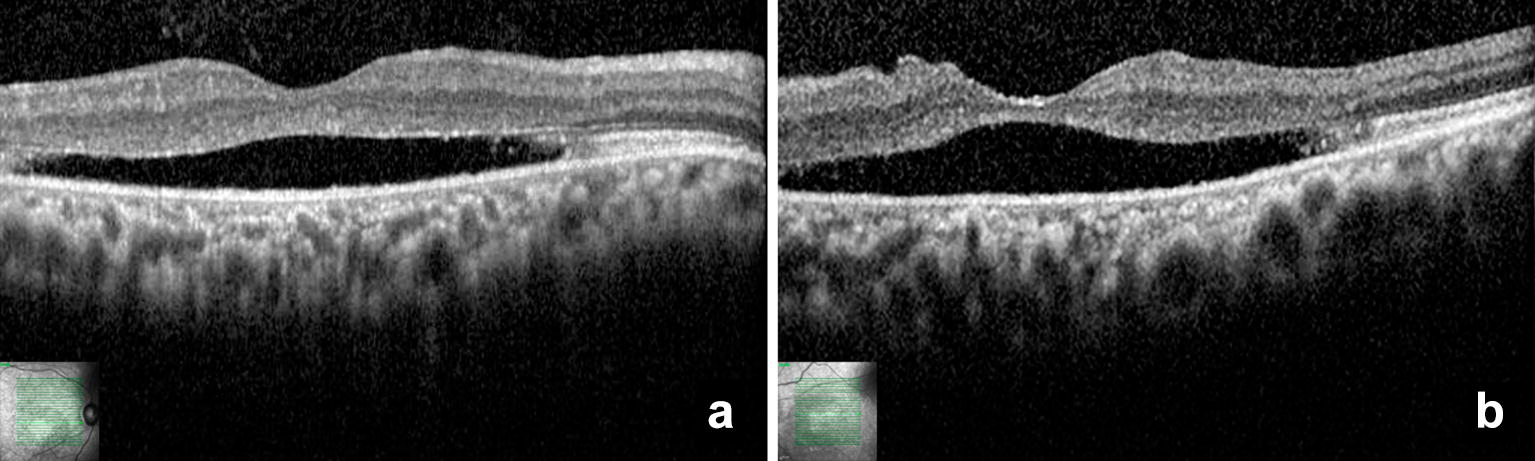
Optical coherence tomography (**a** and **b**) of the patient II-3 in the “vitelliruptive” stage showing neurosensorial retinal detachment, and severe photoreceptor outer segment layer and retinal pigment epithelium atrophy

**Fig. 6:**
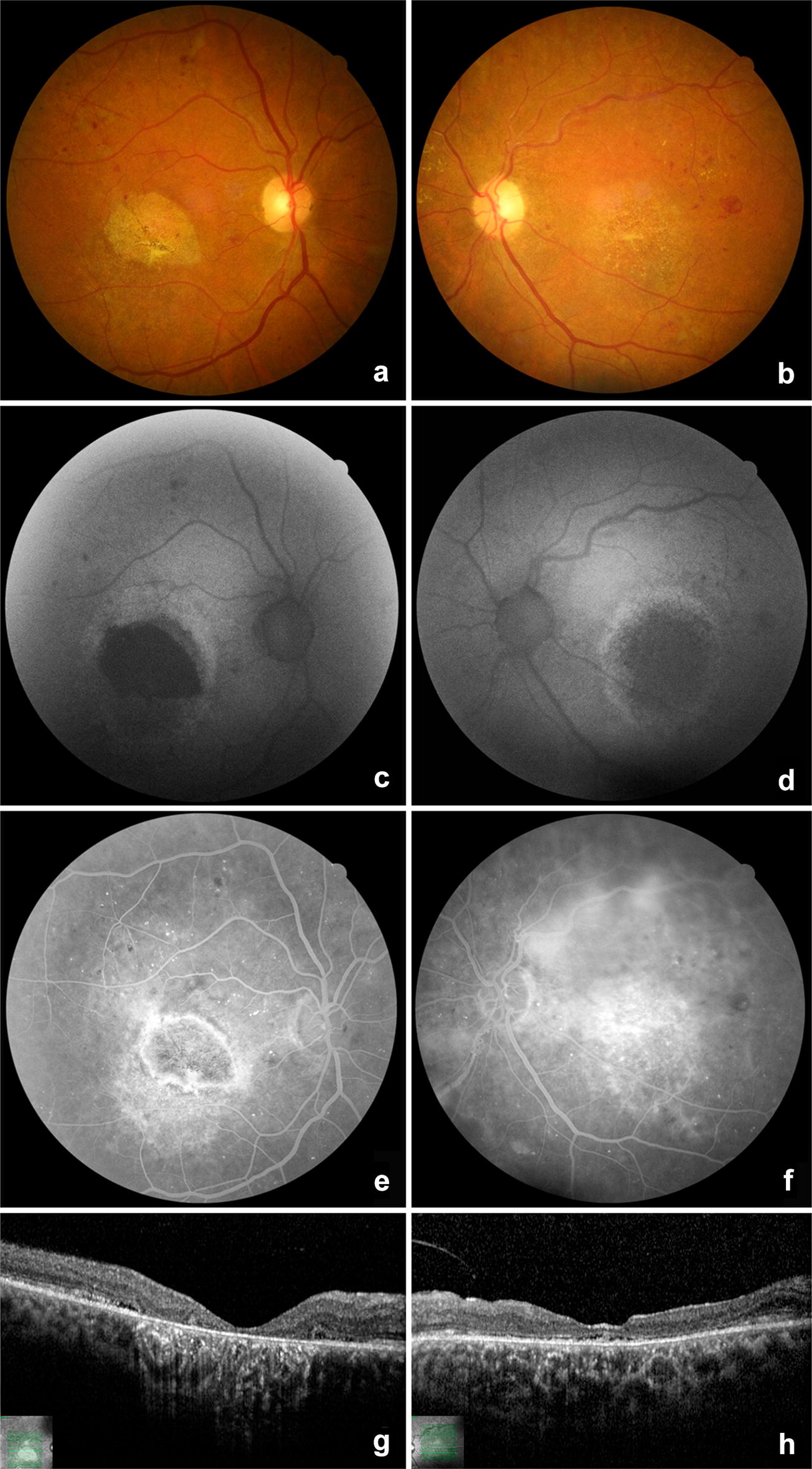
Color fundus photography (**a** and** b**), fundus autofluorescence (**c** and** d**), fluorescein angiography (**e** and** f**) and optical coherence tomography (**g** and** h**) of the patient II-5 in the “atrophic” stage. Both foveal regions are yellowish due to retinal pigment epithelium atrophy, mainly in the right eye, seen as a hypoautofluorescent and hyperfluorescent central region (window defect). There is a marked loss of outer retinal layers, especially in the right fovea

**Fig. 7:**
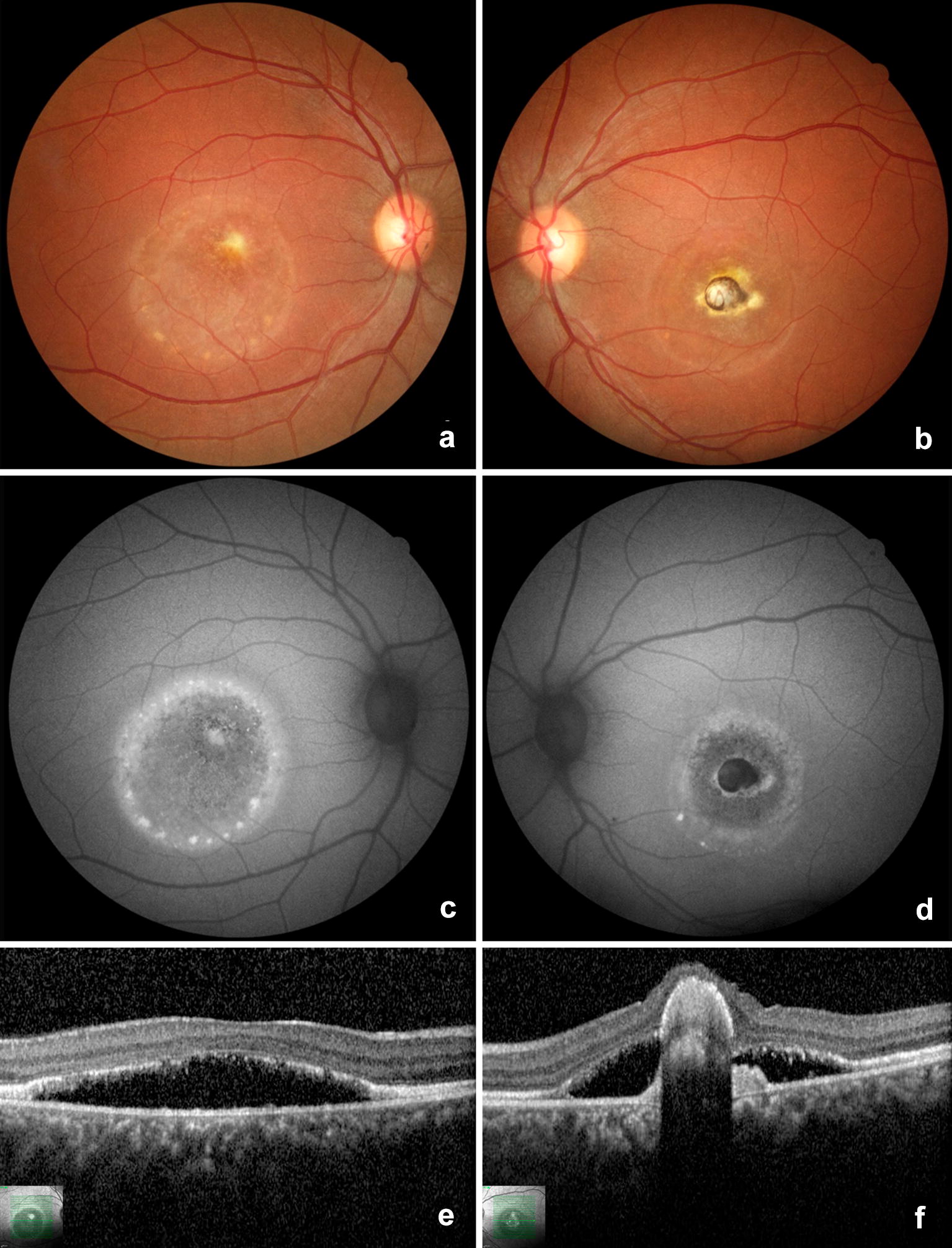
Color fundus photography (**a** and **b**), fundus autofluorescence (**c** and **d**), and optical coherence tomography (**e** and** f**) of the patient III-1 in the “vitelliruptive” stage. The yellowish vitelliform material is seen as a hyperautofluorescent lesion at the fovea and periphery of the retinal detachment. An early foveal disciform scar from fibrovascular proliferation is evidenced as a gray-white lesion in the left eye

**Fig. 8:**
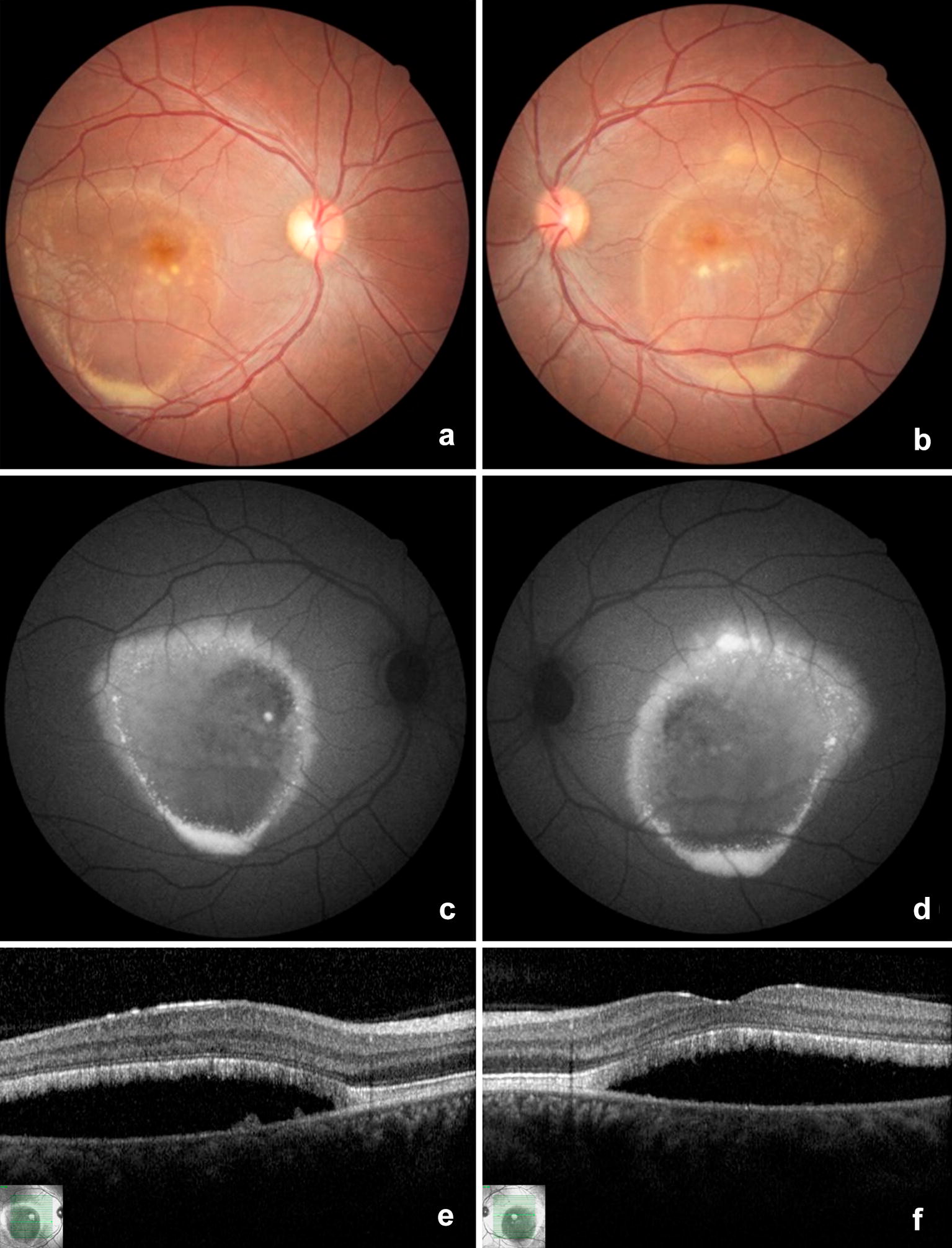
Color fundus photography (**a** and **b**), fundus autofluorescence (**c** and **d**), and optical coherence tomography (**e** and **f**) of the patient III-2 in the “pseudohypopyon” stage. A ring of yellowish hyperautofluorescent vitelliform material accumulation is seen in the margin of the neurosensorial retinal detachment, with inferiorly lipofuscin deposit

A complete family history and investigation of ocular abnormalities were performed. All the members of the family presented a normal slit lamp examination of the anterior chamber, normal cup-to-disc ratio and normal intraocular pressure (values described in Table 1). The family’s ancestry was not established given the great miscegenation in Brazil and the lack of this information by the patients. Ophthalmological examination and complementary exams of the relatives revealed several macular lesions corresponding to different stages of BVMD in other six patients, as shown in Table 1 and Figs. 3, 4, 5, 6, 7 and 8. The EOG of patient III-1 also showed a reduced Arden ratio in both eyes, reinforcing the diagnosis of BVMD. Genetic analysis of the *BEST1* gene was performed in patient III-2 and revealed four heterozygous variants (c.47C > T, c.109T > C, c.482-24C > T, c.1410G > A) and one homozygous variant (c. 636 + 44C > T), confirming the BVMD diagnosis.

**Table 1:** Patients characteristics and summary of macular findings

Patient	Age	Eye	BCVA	SEQ	IOP	Disease stage	YSD	RPEa	SRD	SRFi	FPOSa
II-1	74	OD	20/60	0	14	Stage V		✓		✓	
		OS	CF at 50 cm	0	15	Stage V		✓		✓	✓
II-2	72	OD	20/200	+ 5.50	14	Stage IV		✓	✓		✓
		OS	20/160	+ 5.50	12	Stage IV		✓	✓		✓
II-3	62	OD	CF at 50 cm	+ 2.00	16	Stage IV		✓	✓		✓
		OS	CF at 50 cm	+ 2.75	16	Stage IV		✓	✓		✓
II-5	57	OD	20/125	+ 1.25	16	Stage V		✓			✓
		OS	20/200	+ 0.50	16	Stage V		✓			✓
II-7	54	OD	20/25	+ 1.00	13	Stage III	✓	✓	✓		
		OS	20/25	+ 1.50	12	Stage III	✓	✓	✓		
III-1	29	OD	20/25	− 0.50	13	Stage IV	✓		✓		
		OS	20/50	− 0.25	13	Stage IV	✓		✓	✓	✓
III-2	24	OD	20/20	− 0.25	10	Stage III	✓		✓		
		OS	20/20	− 0.50	11	Stage III	✓		✓		

*BCVA* best-corrected visual acuity, *CF* counting fingers, *FPOSa* foveal photoreceptor outer segments atrophy, *IOP* intraocular pressure (mmHg), *OD* right eye, *OS* left eye, *RPEa* retinal pigment epithelium atrophy, *SEQ* spherical equivalent, *SRFi* subretinal fibrosis, *SRD* serous retinal detachment, *YSD* yellow subretinal deposits, *Symbols* ✓ presence of the abnormality

## Discussion

BVMD is a rare hereditary maculopathy, presenting bilaterally with a typically juvenile onset. Despite foveal lesions, affected individuals initially present with normal vision followed by slow loss of central visual field [1]. Genetic testing for the *BEST1* gene is not mandatory in all suspected cases, as fundus appearance, EOG, and family history allow proper diagnosis. However, it is useful to confirm the disease in atypical cases and in probands [7]. Several lesions may mimic the vitelliform phenotype, such BVMD, adult-onset foveomacular vitelliform dystrophy, age-related macular degeneration, central serous chorioretinopathy, acute idiopathic exudative polymorphous vitelliform maculopathy, large cuticular drusen, vitreomacular traction syndrome, and pigment epithelial detachment [8]. Differentiation between these diseases is important due to their different pathogenesis, genetic inheritances, therapeutic approach and prognostic. Multimodal imaging approach may be a helpful tool to access etiological diagnosis.

This report had several limitations, including its cross-sectional design, lacking information about ophthalmological phenotypes of patients I-1 and I-2 (all other members of the family were examined and only patients II-4, -5 and -8 presented a normal ophthalmologic examination), the absence of genetic analysis and the inability to perform all exams in all patients. However, we describe multimodal imaging findings of the largest Brazilian family with BVMD. In this report, we present a two-generation family of seven affected Brazilian individuals with BVMD. EOG performed in patients of each generation (II-7 and III-1) were abnormal. It is possible that individuals classified as “without ocular abnormalities” (Fig. 1) may have subclinical changes on EOG or mutation in *BEST1*.

A variable range of phenotypes is notable (Table 1), with stage IV being the most prevalent. Furthermore, only one individual (III-1) presented significant asymmetry between eyes due to a foveal disciform scar at the left eye (Fig. 7). Multimodal imaging was helpful for diagnosis, to identify patients with high risk of severe bilateral visual impairment, and for patient guidance on ocular lesions. Foveal photoreceptor outer segment and retinal pigment epithelial atrophy were the main fundus abnormalities associated with lower visual acuity. BVMD is also associated with shallow chamber, hyperopia, and glaucoma [1]. Although extrapolating the overall study objective and the absence of multimodal analysis of anterior segment, eight eyes with normal slit lamp examination have a hyperopic spherical equivalent.

There are few studies of Brazilian patients with BVMD, specially using multimodal imaging, and none of them demonstrate the variable clinical presentation in members of the same family and the correlation of clinical data to multimodal imaging findings. To better understand the ophthalmological aspects of BVMD in Brazilian patients, it is important to compare with other reports worldwide. Our findings corroborate others in the international literature that BVMD may have a variable clinical presentation and its correlation with multimodal imaging findings [4, 9].

## Conclusion

This is the largest case series of a Brazilian family with BVMD. Multimodal imaging analysis supported the accurate diagnosis of patients in late stages of BVMD and their asymptomatic relatives in early stages. Loss of photoreceptors outer segments and RPE atrophy are significant late lesions related to severe visual acuity impairment. Ophthalmological evaluation and pedigree analysis of families with BVMD are important to early diagnosis and to perform adequate genetic counseling.

In the future, easier access to multimodal imaging exams and genetic screening tests will be helpful to early diagnosis and treatment in BVMD disease.

## Abbreviations

BVMD: best vitelliform macular dystrophy
EOG: electrooculography
FA: fluorescein angiography
FAF: fundus autofluorescence
OCT: optical coherence tomography
POS: photoreceptor outer segment
RPE: retinal pigment epithelium
